# Osteological and Biomolecular Evidence of a 7000-Year-Old Case of Hypertrophic Pulmonary Osteopathy Secondary to Tuberculosis from Neolithic Hungary

**DOI:** 10.1371/journal.pone.0078252

**Published:** 2013-10-30

**Authors:** Muriel Masson, Erika Molnár, Helen D. Donoghue, Gurdyal S. Besra, David E. Minnikin, Houdini H. T. Wu, Oona Y-C. Lee, Ian D. Bull, György Pálfi

**Affiliations:** 1 Archaeology, University of Edinburgh, Edinburgh, Scotland, United Kingdom; 2 Department of Biological Anthropology, University of Szeged, Szeged, Hungary; 3 Centre for Clinical Microbiology and Centre for the History of Medicine, University College London, London, United Kingdom; 4 School of Biosciences, University of Birmingham, Egbaston, Birmingham, United Kingdom; 5 Organic Geochemistry Unit, School of Chemistry, University of Bristol, Bristol, United Kingdom; Centers for Disease Control and Prevention, United States of America

## Abstract

Seventy-one individuals from the late Neolithic population of the 7000-year-old site of Hódmezővásárhely-Gorzsa were examined for their skeletal palaeopathology. This revealed numerous cases of infections and non-specific stress indicators in juveniles and adults, metabolic diseases in juveniles, and evidence of trauma and mechanical changes in adults. Several cases showed potential signs of tuberculosis, particularly the remains of the individual HGO-53. This is an important finding that has significant implications for our understanding of this community. The aim of the present study was to seek biomolecular evidence to confirm this diagnosis. HGO-53 was a young male with a striking case of hypertrophic pulmonary osteopathy (HPO), revealing rib changes and cavitations in the vertebral bodies. The initial macroscopic diagnosis of HPO secondary to tuberculosis was confirmed by analysis of *Mycobacterium tuberculosis* complex specific cell wall lipid biomarkers and corroborated by ancient DNA (aDNA) analysis. This case is the earliest known classical case of HPO on an adult human skeleton and is one of the oldest palaeopathological and palaeomicrobiological tuberculosis cases to date.

## Introduction

Hypertrophic Osteoarthropathy (HOA), also known as Marie-Bamberger disease, is a periosteal phenomenon characterised by the symmetrical (diffuse or distal) appearance of new bone mainly on the shaft of the long bones. The reaction can result in “appliqué” (new bone with sharply defined edges distinguishable from the underlying bone) or surface form that covers the entire bone with no visible edge. It is extremely rare as a primary pathology and is usually encountered in its secondary form, also known as Hypertrophic Pulmonary Osteopathy (HPO). Today, its most common causes are intrathoracic cancer and chronic intrathoracic infection [1], [2]. However, in the past tuberculosis would have been a more likely cause. Only a few cases of HOA/HPO diagnosis have been reported in the archaeological record. In one of those cases, tuberculosis (TB) was successfully identified as the possible primary cause of HOA/HPO [3]. In their study, Webb and Thomas [4] associated HOA/HPO particularly with severe and untreated pulmonary tuberculosis. In their recent study of a Portuguese population from a pre-antibiotic era, Assis and colleagues [5] found a strong statistical correlation between HOA/HPO and tuberculosis in the skeletal remains.

HPO is a rare find in the archaeological record. The oldest documented cases in Europe include a Merovingian skeleton from the site of Les Rues des Vignes (Nord, France) dated AD500 to 700 [6], and a medieval 40–50 year-old male from Czarna Wielka (Grozish, Poland) [7]. In a collection of one thousand individuals from Pre-Hispanic Mexico, two presented with HOA/HPO [8]: a young female from a Maya site from the Classic period (AD 300 to 900) and a young adult male from the Ticoman site from the Formative period (2000 BC to AD 100). Most recently in the Middle East, the skeletal remains of a 12-month old infant recovered from the underwater Neolithic site of Atlit-Yam, Israel, dated to 9250-8160 BP, were described as showing evidence of HOA, in addition to *Mycobacterium tuberculosis* aDNA and mycolic cell wall biomarkers [9].

Tuberculosis is a disease of infancy, young adults and the elderly. It is important not to restrict the diagnosis of tuberculosis in palaeopathological cases to the modern clinical diagnostic criteria for TB, as skeletal changes may have differed in the past [10]. Classical tuberculosis pathology includes vertebral fusion and collapse leading to Pott’s disease, knee joint ankylosis, hip joint destruction, cold abscess on the sacrum or vertebrae and endocranial TB. Other osseous change probably related to tuberculosis include rib periostitis, hypervascularization, diffuse symmetrical periostitis (HPO), endocranial changes such as *serpens endocrania symmetrica* (SES) and abnormal blood vessel impressions [11]. Rib changes may include sharply demarcated lytic lesions or diffuse periostitis on the ventral side of the ribs, possibly caused by adjacent soft tissue infection. Most rib changes are associated with individuals suffering from pulmonary TB, particularly in the left chest, and although those lesions cannot be considered specifically characteristic of pulmonary tuberculosis, they can indicate a non-specific chronic pulmonary disease, with tuberculosis as the most likely cause [12], [13]. Porotic hyperostoses, such as *cribra orbitalia* and *cribra cranii*, are generally attributed to iron-deficiency anemia, which can develop from the interaction of several factors, such as weaning practices, diet, hygiene, parasites and infectious diseases, so may also be associated with tuberculosis.

The Atlit-Yam study [9] provides the earliest biomolecular evidence of tuberculosis in humans. Both DNA and lipid biomarkers analyses confirmed that the 25-year old female and the 12-month old infant were infected with a human lineage of the *Mycobacterium tuberculosis* complex. The osteological pathological evidence was very scarce on the adult female. In the infant, it consisted of endocranial changes (SES) and periostitis on tubular bones, consistent with tuberculosis. Although the periostitis was described as HOA, there is no evidence of symmetry of lesions. Prior to this study, the oldest recognised cases of tuberculosis came from Neolithic Europe. A 15-year old juvenile and a 30-year old female from Liguria, Italy, dating from the Middle Neolithic in the first half of the 4^th^ millennium BC, were both diagnosed on the basis of spinal osteolytic lesions [14], [15]. Another probable case originated from Zlota, Poland, based on the spine of a Neolithic male [16]. Tuberculosis has also been confirmed previously by DNA analyses in pre-dynastic Egyptian skeletons (3500-2650 BC), both with bony changes [17] and without [18]. In Hungary, Pott’s disease in an adult male, dating from the Late Neolithic/Early Copper Age (5^th^ millennium BC) was discovered recently at the site of Alsónyék-Bátaszék [19]. This has not yet been confirmed by molecular biomarkers, but the morphological observations unequivocally indicate an advanced stage of vertebral tuberculosis. Several other possible tuberculosis cases have been discovered recently from the 5000 year-old site of Vésztő-Mágor, Hungary, associated with archaeological material from the Tisza Culture [20]. Palaeomicrobial analysis of the dental pulp region in the teeth of one of the cases confirmed the presence of *M. tuberculosis* aDNA [21].

The present study was based on human skeletal remains from the Neolithic tell settlement of Hódmezővásárhely-Gorzsa in the South of Hungary. Macroscopic analyses revealed a widespread symmetrical periostitis on the long bones and the ribs of a young adult male, indicating a case of HPO. The strong association with tuberculosis, as described above, made further biomolecular studies of this 7000 year-old skeleton imperative to ascertain the presence of tuberculosis at the Tisza Culture site. As noted above [9], [22], the detection of aDNA and lipid biomarkers can offer confirmation of the presence of tuberculosis in archaeological material, so there was good expectation of finding such biomarkers in HGO-53. In addition, the mycocerosic and mycolipenic acid cell wall lipid biomarkers appear to be more stable, and can thus offer conclusive support as demonstrated in a very ancient, 17,000 year-old bison metacarpal [23].

### Archaeological Background

**Figure 1:**
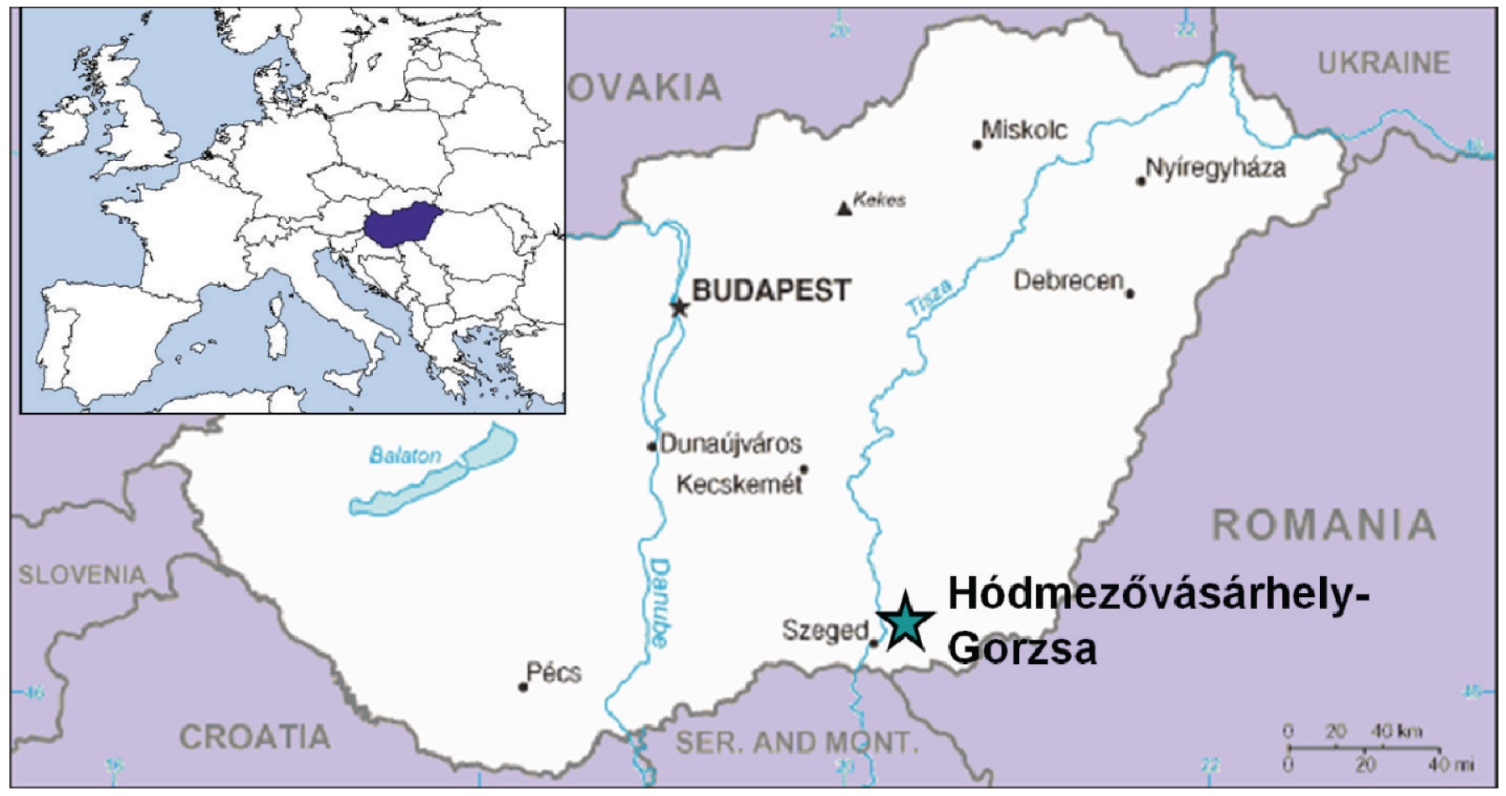
Location of the site. The Late Neolithic Tell settlement of Hódmezővásárhely-Gorzsa, located in the South of Hungary, about 15 miles North East of Szeged and 9 miles South West of Hódmezővásárhely in the Tisza-Maros angle. Inset shows general geographic location.

The Late Neolithic Tell settlement of Hódmezővásárhely-Gorzsa is located in the South of Hungary, about 15 miles North East of Szeged and 9 miles South West of Hódmezővásárhely in the Tisza-Maros angle (Fig. 1). It had been on a natural elevation surrounded by streams and marshes, and was occupied through six settlement phases starting from the Early Tisza culture. Only two percent of the site has been investigated to date. The site was initially investigated by Gazdapusztai between 1955 and 1957 [24], [25], [26], and excavations were undertaken by Horváth between 1978 and 1996 [27], [28], [29], [30].

The settlement phase of the Tisza Culture occurred during the first half of the fifth millennium BC, with an occupation time span of at least 300 years. Radiocarbon analysis of twenty samples from the site date this settlement to 4970 - 4594 BC [31], [32], [33] or 4850 - 4550 cal BC [34], [35] with a 68.3% confidence interval. These dates were recalibrated by Masson (unpublished PhD Thesis, 2013, University of Edinburgh) using the calibration curve IntCal04 for Northern Hemisphere [36] in the dating programme OxCal 4.1 [37]. The original uncalibrated dates by Hertelendi & Horváth [33] yielded results ranging from 4932 to 4602 BC with 95.4% confidence interval after recalibration. This occupation span fits with overall ranges for the Tisza culture [34], [35] and the Hungarian Late Neolithic [38], 4970–4490 BC and 4970–4380 BC respectively. Using new recalibrations, Yerkes and colleagues [39] utilised 107 Late Neolithic samples to produce a range of dates from 5021 to 4402 BC for the whole period.

The human skeletal remains recovered from Hódmezővásárhely-Gorzsa are housed in the collection of the Biological Anthropology Department of the University of Szeged, on loan from the Móra Ferenc Múzeum in Szeged. No permits were required for the described study, which complied with all relevant regulations. Access to the collection was granted by both Móra Ferenc Múzeum and the Biological Anthropology Department of the University of Szeged. Seventy-one individuals were recovered in total from the Tisza (Late Neolithic) Culture, including 56 who had been buried in graves within the settlement and the partial remains of a further possible fifteen recovered from pits, ditches, houses and as stray finds. Juveniles accounted for a third of the remains. Of the adult remains where sex could be determined, two-thirds were female. Pathological analyses seemed to indicate that this population had been mostly non-violent, leading a physically stressful life, prone to infections and with a high rate of dental disease [40].

Unfortunately, there are no published maps of the site, and there is no information currently available on the location of the graves and other remains in relation to the settlement and to each other. However, recent radiocarbon analysis at the Herlendi AMS C-14 Lab in Debrecen, Hungary (AMS Lab code DeA-2485.1.1), on bone fragments from HGO-53 confirmed that this individual dated back to the start of the fifth millennium BC, with a calibrated age range of 4780–4715 BC with 1 sigma, based on HGO-53 radiocarbon age of 5872±32 BP and the intcal09.14c calibration data set [41].

## Materials and Methods

### Morphological Analysis

The remains of HGO-53, the skeleton from grave 64, were very fragmentary with over one thousand fragments, though his skeleton was mostly complete. The examination was carried out macroscopically at the Biological Anthropology Department of Szeged University. The palaeopathological analysis based on macromorphological observations [42], [43] was undertaken at the same laboratory.

Sex was estimated based on several morphological methods. Both skull and pelvis indicated that this individual was a male. Bone dimensions also reflected a male individual. Skeletal and dental development aged this individual to around 19–20 years old. Stature was estimated based on long bone lengths to 165 cm ±4 cm. See Document S1 for full details of the methodologies used in estimating age, sex and stature of HGO-53.

### *M. Tuberculosis* aDNA Analysis

The recommended protocols for aDNA were followed. Approximately 55 mg of bone powder was taken from each sample of a rib, tibia and vertebra. The DNA was extracted as described previously [9], [44]. PCR was used to amplify any DNA from specific regions of the multicopy IS*6110* and IS*1081* regions of the *M. tuberculosis* complex. Amplified DNA was examined initially by agarose gel electrophoresis [45]. Subsequently, these primers were used on a Real-Time platform, to enable the detection of DNA using SYBR Green and melt analysis. Sequencing was attempted after extraction of DNA from gel slices. See Document S2 for full details of the methodologies used in the aDNA analysis.

### Lipid Biomarker Analysis

Lipid biomarkers from a rib sample of HGO-53 (556 mg) were extracted, derivatised and fractionated, as described previously [9], [23]. See Document S3 for full details of the methodologies used in the lipid biomarker analysis.

## Results

### Macroscopic Analysis

**Figure 2:**
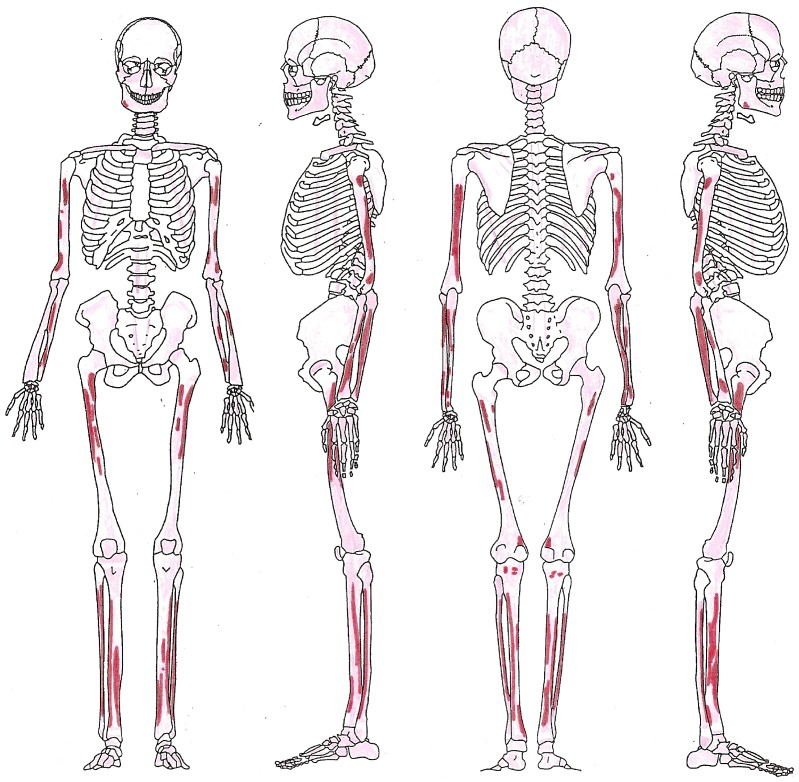
HGO-53 - Location of periostitis. The strikingly symmetrical diffuse periostitis on the bones of this young adult male revealed by the morphological analyses is a characteristic sign of Secondary Hypertrophic Osteoarthropathy (HOA).

**Figure 3:**
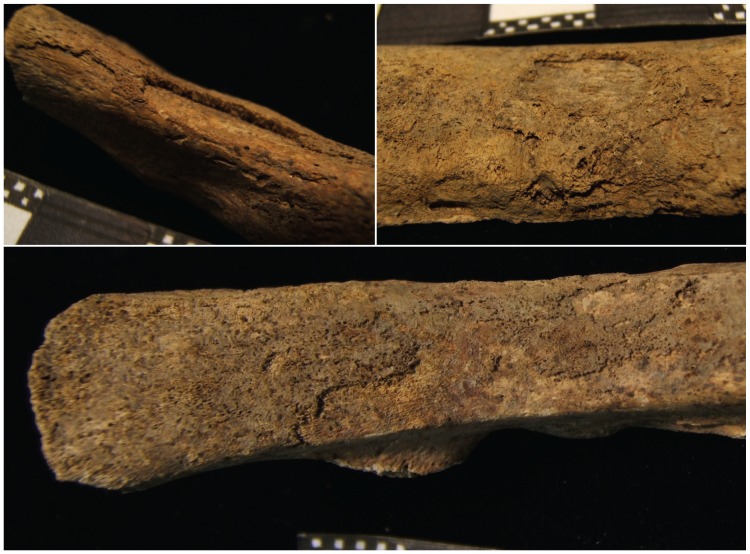
HGO-53– Ribs. Active diffuse periostitis with extensive bone formation visible on the ribs.

**Figure 4:**
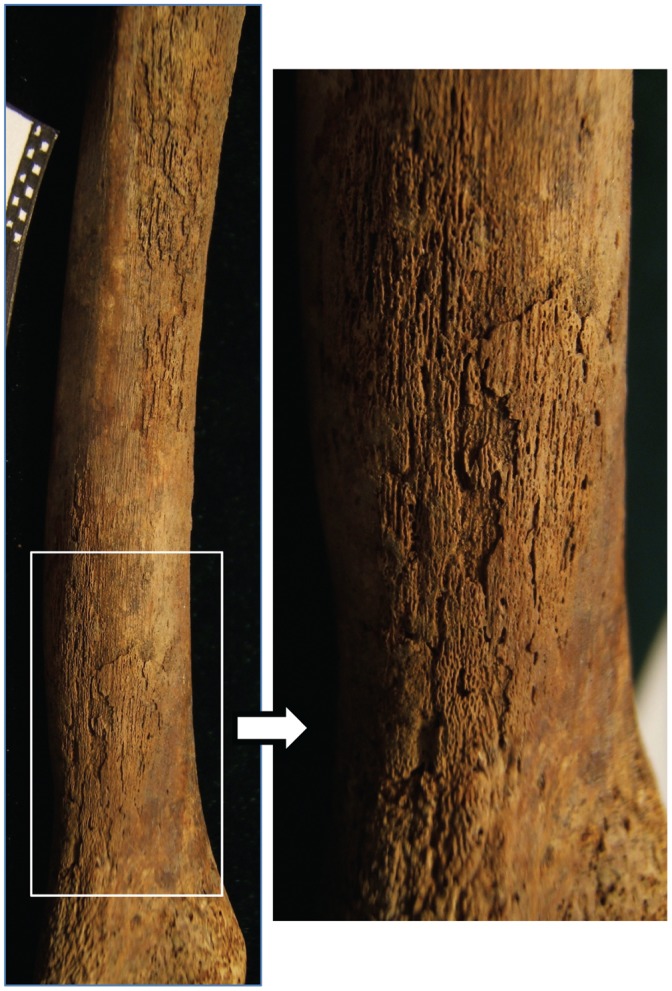
HGO-53– Upper Limbs. Active diffuse periostitis on distal end of the ulna.

**Figure 5:**
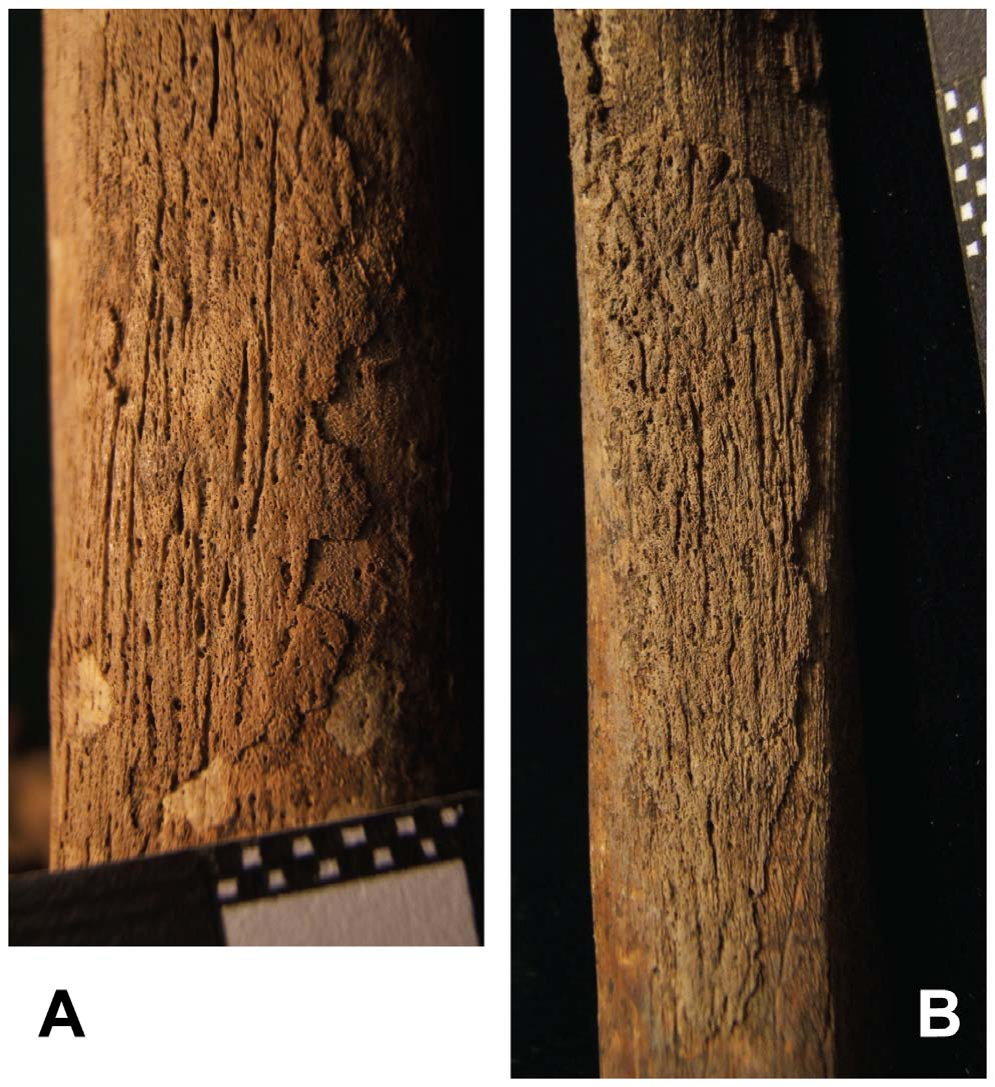
HGO-53– Lower Limbs. “Appliqué” periostitis on femur (a.) and fibula (b).

**Figure 6:**
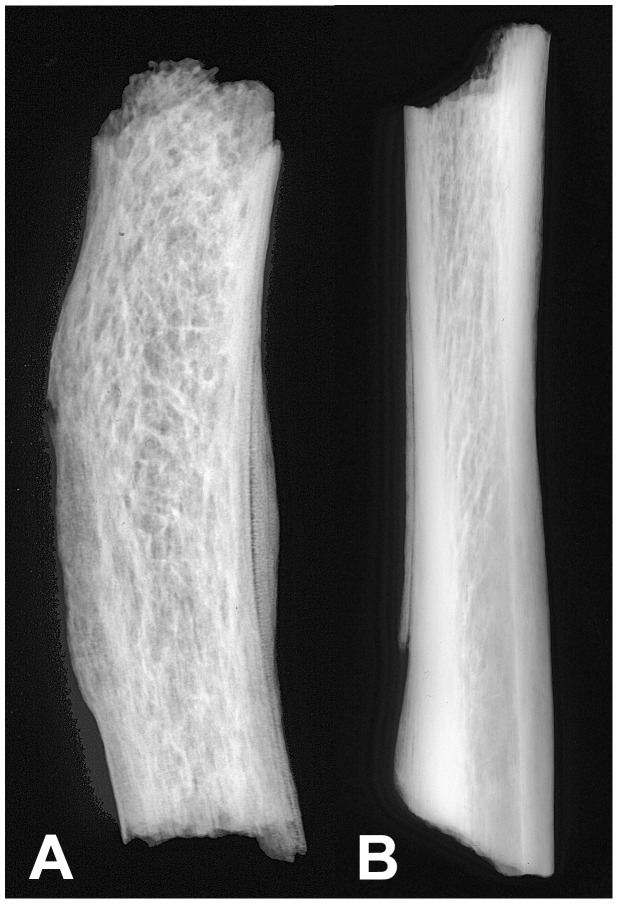
HGO-53– Radiographs. “Appliqué” periostitis on a fragment of rib (A) and a fragment of fibula (B).

Pathology was observed on the skull, thorax, shoulder, upper limbs, spine, lower limbs and feet of HGO-53 (Fig. 2). Light *cribra orbitalia* and *cribra cranii* were visible on the skull, and a small area of periostitis was visible on the mandible. Cavitations were observed on fragments of vertebral bodies. Active diffuse periostitis with severe bone formation on the ventral surface of the heads of left ribs was observed, although none on the heads of right ribs. Unsided fragments of ribs also showed active diffuse periostitis, with a focal lytic lesion accompanied by reactive surface new bone formation in one case (Fig. 3). All long bones presented evidence of widespread active periostitis with woven bone formation, mostly along their shafts and strikingly symmetrical both on the upper limbs (Fig. 4) and the lower limbs (Fig. 5). Signs of periostitis were also visible on the foot bones of both sides. See Document S1 for a detailed description of HGO-53 skeletal pathologies, and figure 6 for the radiographs of a rib fragment and a fragment of fibula from HGO-53, clearly showing the new bone formation along both shafts.

The strikingly symmetrical diffuse “appliqué” periostitis on the bones of this young adult male revealed by the morphological analyses is a characteristic sign of Hypertrophic Pulmonary Osteopathy (HPO). This strongly indicates that this individual had suffered from a chronic pulmonary disease. In addition, the analysis revealed distinctive changes on the ribs of the left chest, cavitations in the vertebral bodies and signs of porotic hyperostosis. Considering all of this evidence, together with the association of HPO with tuberculosis (especially in its severe untreated form), and the age of this young man, it is likely that this individual had pulmonary tuberculosis. Based solely on the pathology, however, all that can be stated with certainty is that HGO-53 is one of the earliest cases of chronic pulmonary disease in the archaeological record. Due to the antiquity of this population and the importance this case has for palaeopathology, it was decided to carry out the biomolecular analyses.

### aDNA Analysis

DNA was recovered from HGO-53 but was very unstable, due to the condition of the skeletal remains. The sample of vertebra from HGO-53 was positive for PCR using primers specific for *M. tuberculosis* IS*1081*, with an amplicon of 113 bp (Document S4). Bands of the appropriate size were excised from gels and a DNA purification protocol followed. However, sequencing was unsuccessful. The DNA extractions were repeated and examined on the Real-time platform. Again the vertebral sample was positive for IS *1081* shown by melt analysis (Document S4). However, no positive results were obtained using primers for IS *6110*. The tibia and rib samples were negative.

### Lipid Biomarkers Analysis

**Figure 7:**
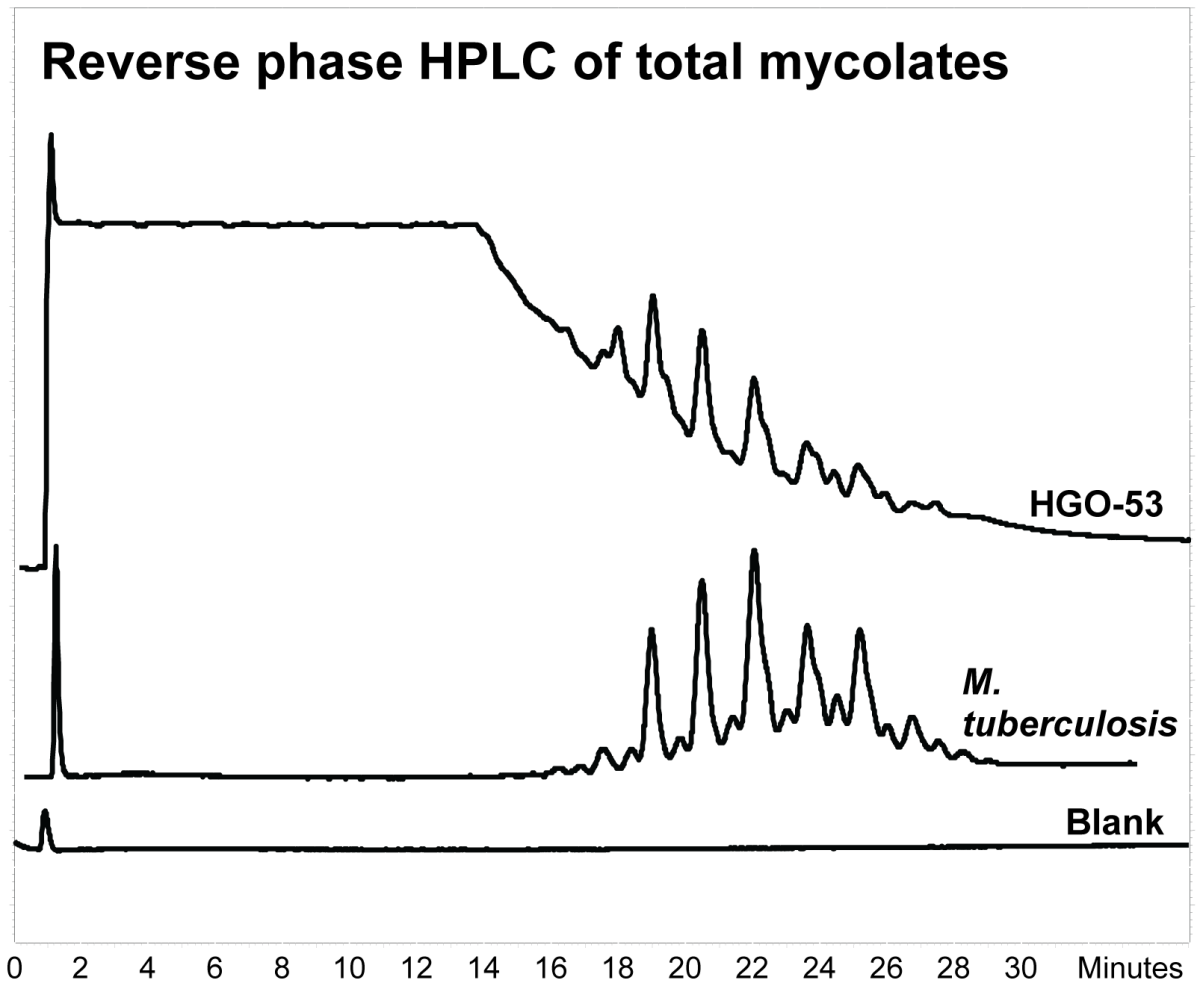
HGO-53– Profile of total mycolic acids. Reverse phase fluorescence HPLC of pyrenebutyric acid derivatives of pentafluorobenzyl esters of total mycolic acids from HGO-53 and standard *M. tuberculosis*.

**Figure 8:**
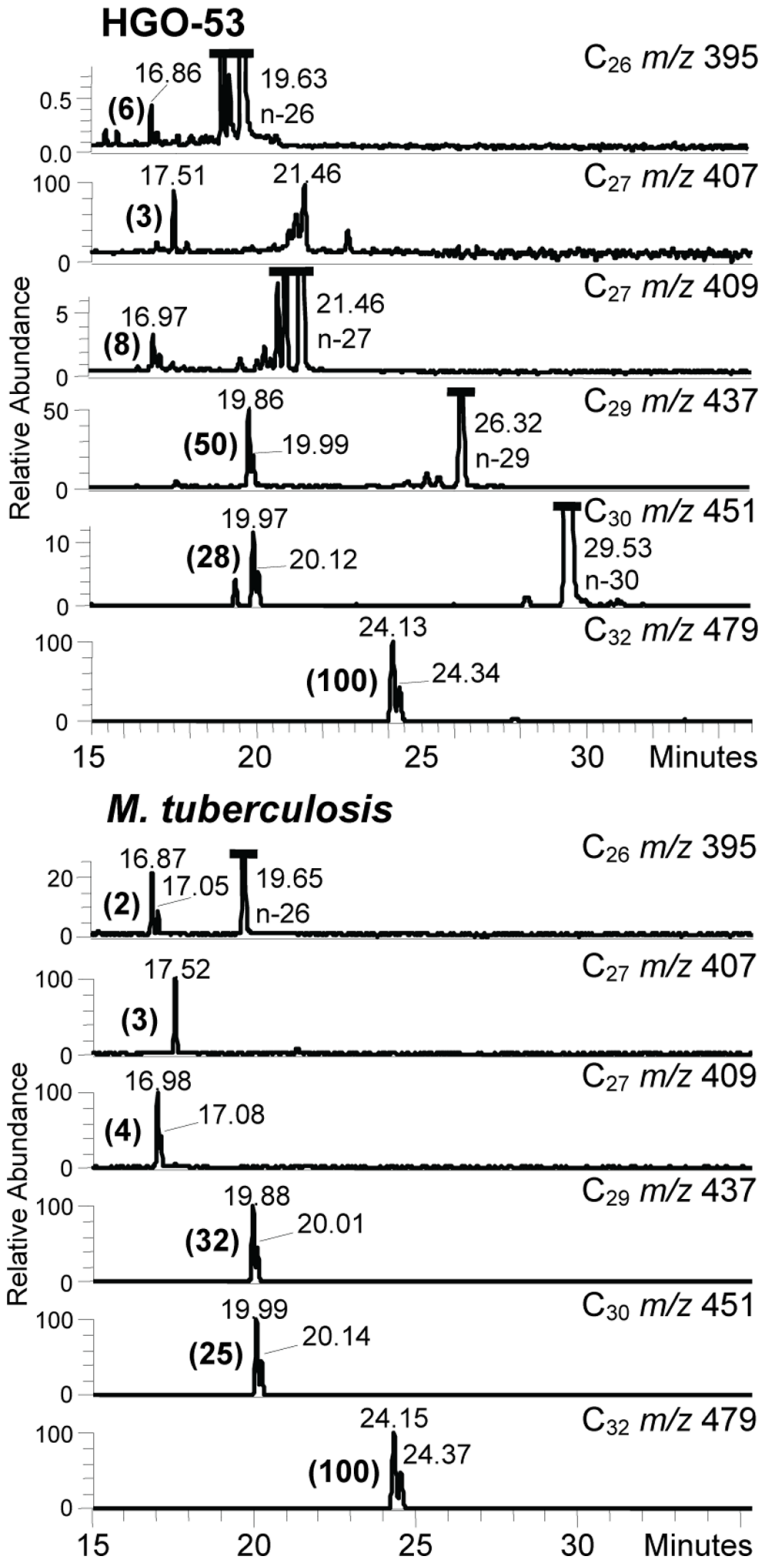
HGO-53– Profiles of mycolipenic and mycocerosic acids. Selected ion monitoring NI-CI GC-MS of mycolipenic and mycocerosic acid pentafluorobenzyl fractions from HGO-53 and standard *M. tuberculosis*. The C_27_ *m/z* 407 ion is for C_27_ mycolipenate; ions at *m/z* 395, 409, 437, 451 and 479 are for mycocerosates. The intensities of the mycocerosate and mycolipenate peaks, in brackets, are normalized to that (100) of the major C_32_ mycocerosate. In the HGO-53 profiles, the peaks with retention times 19.63, 21.46, 26.32, and 29.53 correspond to 26, 27, 29 and 32 carbon straight-chain acids, respectively.

Reverse phase HPLC of the pyrenebutyrate- pentafluorobenzyl (PBA-PFB) mycolate fractions indicated the presence of long-chain mycolic acids in the bone sample from HGO-53 (Fig. 7). The rather weak profile correlated with the standard profile for *M. tuberculosis.* However, normal phase HPLC of the total mycolate fraction gave only a small peak for α-mycolates, indicating that any methoxy- or ketomycolates had been degraded (data not shown). In contrast, the NI-CI GC-MS profiles (Fig. 8) of mycocerosic and mycolipenic acids provided confirmation of tuberculosis. The mycocerosates are recognisable by their appearance as double peaks following racemisation, but the C_27_ mycolipenates (Fig. 8, *m/z* 407) are clear single peaks as they are unable to racemise [23], [46].

## Discussion

The DNA analysis was undertaken in the former Department of Medical Microbiology at University College London, which has considerable experience of working with aDNA to study tuberculosis in the past [44], [47], [48], [49], [50]. It is well-known that DNA is not a stable molecule and degrades with age [49], although the successful DNA analysis of the Atlit-Yam remains [9] demonstrates the importance of local environmental conditions at the site. Clearly, the Hódmezővásárhely-Gorzsa site was not especially conducive for aDNA preservation, so no confirmatory analysis was possible. The preliminary finding of *M. tuberculosis* complex aDNA in the IS*1081* region, but not that of IS*6110*, is probably due to chance but may also be influenced by copy number. There are six copies of IS*1081* in every member of the *M. tuberculosis* complex. However, the copy number of IS*6110* varies between strains and today may even be absent, although not in European isolates. The range is from 1 to 24 copies per cell in human *M. tuberculosis* but *M. bovis* always has a low copy number (1–5). It is possible that the infection was caused by *Mycobacterium bovis*, but the DNA preservation was too poor to enable this to be determined. However, in the literature human tuberculosis caused by *M. bovis* is extremely rare [51].

As an alternative to aDNA biomarkers for ancient tuberculosis, Gernaey and colleagues [52], [53] introduced the complementary use of mycolic acids. These robust lipid biomarkers do not suffer as much from contamination problems, as the sensitive methods used involve no amplification. This now established technique has already been refined several times to ensure maximum potential [9], [23]. Redman and colleagues [46] demonstrated that mycocerosic and mycolipenic acid biomarkers are also robust indicators of tuberculosis in ancient remains. All these classes of lipid biomarkers are totally distinct from anything found in mammalian tissue and they provide good diagnoses for members of the *M. tuberculosis* complex.

Reverse phase HPLC of the total mycolic acid fraction (Fig. 7) provided a very weak profile in the same region as that for the *M. tuberculosis* standard. Although some of the peaks in the HGO-53 extract correlated with those in the standard, it is apparent that some degradation had taken place. The total mycolate profile (Fig. 7) is an overlapping composite of the three characteristic α-, methoxy- and ketomycolic acid types characteristic of *M. tuberculosis,* which can be separated by normal phase HPLC [9], [23]. However, the small amount of material recovered from the reverse phase isolation of the total mycolates from HGO-53 only provided a small signal for α-mycolates on normal phase HPLC (data not shown). This preferential diagenetic decay of the oxygenated methoxy and ketomycolates is in accordance with previous findings, particularly that for a 17,000 year old bison specimen [23]. The mycolate analysis indicates a mycobacterial presence, but it is not conclusive for members of the *M. tuberculosis* complex.

A much more definitive diagnosis of tuberculosis infection was provided by the NI-CI GC-MS investigation of mycocerosic and mycolipenic acid profiles (Fig. 8), which shows a good correlation of the extract from HGO-53 and standard material. In particular, the major C_32_ mycocerosate and the C_27_ mycolipenate are very characteristic for *M. tuberculosis* [22], [23], [46], [54]. The mycocerosic acids are components of exceptionally hydrophobic stable phthiocerol dimycocerosate waxes [54], which might be expected to resist diagenesis better than more highly functionalised mycolic acids. Similarly, but to a lesser extent, the C_27_ mycolipenate is a constituent of relatively apolar pentaacyl trehalose glycolipids [54], which again are relatively hydrophobic.

The lipid biomarker profiles of extracts of the 7000 year old HGO-53 are reminiscent of those recorded for a 17,000 year old extinct bison metacarpal from Natural Trap Cave, Wyoming. Both examples had weak traces of mycolic acids, showing severe degradation. It is apparent that the mycocerosate and mycolipenate biomarker fatty acids are much more resistant to diagenesis than the mycolic acids. However, the mycocerosate/mycolipenate profiles for HGO-53 (Fig. 8) are relatively weaker than those for Natural Trap Bison [23]. For HGO-53, relatively high proportions of indigenous straight-chain C_26_, C_27_, C_29_, and C_30_ fatty acids (Fig. 8) are indicative of the weakness of the extract. It should also be noted that the 556 mg HGO-53 sample is much larger than that (13 mg) used for the ancient bison. Indications are, therefore, that the mycocerosic and mycolipenic acids are particularly robust biomarkers, with potential to help detect tuberculosis of great antiquity.

## Conclusions

This study presents a new case of HPO to enrich the sparse archaeological record of this disease, particularly in prehistoric times. This case is the earliest occurrence of fully-developed HPO on an adult human skeleton to date, confirming the presence of this pathology already in Neolithic Europe. With the successful combination of different scientific methods, including morphological observations and palaeomicrobiological analyses, we were also able to conclusively verify the presence of the *Mycobacterium tuberculosis* complex in Neolithic Europe, as early as 7000 years ago.

## Supporting Information

Document S1 **Detailed results of HGO-53 macroscopic analysis.**
(PDF)

Document S2 **Detailed information on the aDNA methodologies.**
(PDF)

Document S3 **Detailed information on the lipid biomarker analysis.**
(PDF)

Document S4 **Results of aDNA analysis - gels and melt.**
(PDF)
