# Supplementary material for: Osteological and Biomolecular Evidence of a 7000-Year-Old Case of Hypertrophic Pulmonary Osteopathy Secondary to Tuberculosis from Neolithic Hungary
Source: PLoS One. 2013 Oct 30;8(10):e78252. doi: 10.1371/journal.pone.0078252 (PMC3813517; doi:10.1371/journal.pone.0078252)
Supplement: Document S1 — Detailed results of HGO-53 macroscopic analysis. (PDF) [file pone.0078252.s001.pdf]

S1: Detailed Results of Macroscopic Analyses – HGO 53

1. Sex

Sex was estimated based on several morphological methods [1,2,3,4,5,6]. Both skull (nuchal crest, mastoid process, mental eminence, mandible ramus flexure) and pelvis (greater sciatic notch, composite arch, proportion, symmetry and AP line) indicated that this individual was probably a male. Bone dimensions also reflected a male individual.

2. Age

Age was estimated from skeletal and dental development. State of epiphyseal fusion [7,8] was particularly useful in this case, ageing this individual to around 19-20 years old. All long bones, metacarpals, metatarsals, and phalanges were fully fused as well as the glenoid cavity; the ischium still had a line of fusion visible; the iliac crest and vertebral ring epiphyses were fusing; the sternal epiphyseal plate was fusing at the clavicular articulation and very billowy at the first costal notch; and importantly both proximal and distal clavicular epiphyses were still unfused. Third molars had not erupted, although their crowns were fully formed. Morphology of the pubic symphysis [9,10], and the auricular surface [11] was in Phase 1, as was this individual's tooth wear [12,13]. Ageing based on the acetabulum [14] also concurred with an age estimation in the early 20s.

3. Stature

Stature was estimated based on long bone lengths [15] to 165cm ± 4cm (average male height in this population was 168cm, with a female average height of 153cm).

4. Pathology

Light *cribra orbitalia* and *cribra cranii* were visible on the skull, and a small area of periostitis was visible on the right ramus of the mandible, on the buccal side towards the gonial angle. The only dental pathology was evidence of calculus.

Active diffuse periostitis with severe bone formation on the ventral surface of the heads of nine left ribs was observed, although none on the heads of right ribs. Sixty-three unsided

fragments of ribs also showed active diffuse periostitis on all fragments with a focal lytic lesion accompanied by reactive surface new bone formation in one case.

An enthesopathic lesion at the site of the costo-clavicular ligaments was observed on the medial end of the right clavicle. Cavitations (Schmorl's nods or lytic lesions) were observed on six fragments of vertebral bodies.

The left and right humeri also presented with periostitis along the shaft, mainly on the posterior side, with bone destruction and periostitis on the crest of the lesser tubercle. Periostitis was observed along the entire shaft of the left radius, though scarce along the anterior side. The active woven bone formation was mainly located towards the middle of the shaft on the posterior side. The right radius also showed periostitis along the shaft, mainly on the posterior side, with an active woven bone formation. Widespread active periostitis was visible along the shaft of both left and right ulnae, mainly on the posterior side and specifically the lateral half.

Both femurs had widespread active periostitis along the shaft, mainly on the lateral side, with woven bone formation. Both tibiae showed widespread active periostitis along the shaft with woven bone formation. Widespread active periostitis with woven bone formation was also observed on both fibulae, mainly along the medial side.

Slight periostitis below the sustentacular sulcus was visible on the left calcaneus, with a cavitation between the posterior and middle talar articular surface next to the medial edge. The right calcaneus also presented evidence of periostitis below the sustentacular sulcus. Periostitis was observed on the first, fourth and fifth metatarsals on both sides and on the right second metatarsal. The first left proximal phalanx had periostitis on the shaft and a slight cavitation in the metatarsal articular surface, and the first right proximal phalanx had a stronger cavitation on the metatarsal articular surface.

5. References

1. Ferembach D, Schwidetzky I, Stloukal M (1980) Recommendations for age and sex diagnoses of skeletons. Journal of Human Evolution 9: 517-549.
2. Buikstra JE, Ubelaker DH, editors (1994) Standards for Data Collection from Human Skeletal Remains. Fayetteville, Arkansas: Arkansas Archaeological Survey Report Number 44.
3. Bass WM (1995) Human Osteology : A Laboratory and Field Manual. Columbia: Missouri Archaeological Society, Inc.

4. White TD, Folkens PA (2005) The Human Bone Manual. San Diego: Elsevier Academic Press.
5. Bruzek J (2002) A Method for Visual Determination of Sex Using the Human Hip Bone. American Journal of Physical Anthropology 117: 157-168.
6. Loth SR, Henneberg M (1996) Mandibular ramus flexure: a new morphologic indicator of sexual dimorphism in the human skeleton. American Journal of Physical Anthropology 99: 473-485.
7. Scheuer L, Black S (2004) The Juvenile Skeleton. London: Elsevier Academic Press.
8. Schaefer MC, Black S, Scheuer L (2009) Juvenile Osteology: A Laboratory and Field Manual: Academic Press. 384 p.
9. Todd TW (1920) Age changes in the pubic bone. American Journal of Physical Anthropology 3: 285-339.
10. Brooks ST, Suchey JM (1990) Skeletal age determination based on the os pubis: A comparison of the Acsádi-Nemeskéri and Suchey-Brooks methods. Human Evolution 5: 227-238.
11. Lovejoy CO, Meindl RS, Pryzbeck TR, Mensforth RP (1985) Chronological Metamorphosis of the Auricular Surface of the Ilium: A New Method for the Determination of Adult Skeletal Age at Death. American Journal of Physical Anthropology 68: 15-28.
12. Smith BH (1984) Patterns of Molar Wear in Hunter-Gatherers and Agriculturalists. American Journal of Physical Anthropology 63: 39-56.
13. Brothwell DR (1989) The relationship of tooth wear to aging. In: İşcan MY, editor. Age Markers in the Human Skeleton. Springfield: Charles C Thomas. pp. 303-318.
14. Rissech C, Estabrook GF, Cunha E, Malgosa A (2006) Using the Acetabulum to Estimate Age at Death. Journal of Forensic Sciences 51: 213-229.
15. Sjøvold T (1990) Estimation of stature from long bones utilizing the line of organic correlation. Human Evolution 5: 431-447.
