# Supplementary material for: Osteological and Biomolecular Evidence of a 7000-Year-Old Case of Hypertrophic Pulmonary Osteopathy Secondary to Tuberculosis from Neolithic Hungary
Source: PLoS One. 2013 Oct 30;8(10):e78252. doi: 10.1371/journal.pone.0078252 (PMC3813517; doi:10.1371/journal.pone.0078252)
Supplement: Document S2 — Detailed information on the aDNA methodologies. (PDF) [file pone.0078252.s002.pdf]

S2. Detailed information on the aDNA methodologies

Disaggregation of samples and DNA extraction

Skeletal samples were examined for the presence of DNA from the *M. tuberculosis* complex. Recommended protocols for ancient DNA (aDNA) work were followed (O'Rourke et al., 2000; Taylor et al., 2010), with separate rooms and equipment for different stages of the process. Each sample was crushed using a sterile pestle and mortar, weighed and added to 400µl of Proteinase K/EDTA. The slurry was incubated at 56 ^{o}C and mixed on a bead beater daily.

When the sample was solubilised, it was divided and one aliquot treated with 40µl of 0.1 mol^{-1} N-phenacylthiozolium bromide (PTB), to cleave any covalent cross-links thus enabling DNA strand separation and amplification (Poinar et al., 1998). Initially, DNA extraction was carried out according to the protocols described previously (Spigelman et al., 2002) based on initial demineralization, guanidium thiocyanate-based lysis buffer (Boom et al., 1990) and capture onto silica (Höss & Pääbo, 1993). Thereafter, the protocol was modified. Sample tube contents were transferred into separate 9ml tubes of NucliSens® (bioMérieux) lysis buffer containing 5mol^{-1} guanidium thiocyanate and incubated for 1–3 days at 56 ^{o}C. To complete the disruption of bone and any mycobacterial cell wall remnants, samples were boiled, then snap-frozen in liquid nitrogen and thawed in a 65 ^{o}C waterbath. This procedure was repeated twice. Sample tubes were centrifuged at 5000g for 15 min at 5 ^{o}C and the supernatants carefully removed into clean, sterile tubes. DNA was captured by adding 40µl silica suspension (NucliSens®) and mixing on a rotator wheel for 1 h. Tube contents were centrifuged and silica pellets washed once with wash buffer (NucliSens®), twice with 70% (v/v) ethanol (−20 ^{o}C) and once with acetone (−20 ^{o}C). After drying in a heating block, DNA was eluted using 60µl elution buffer (NucliSens®), aliquoted and used immediately or stored at −20 ^{o}C. Silica supernates (500µl) from PTB-negative samples were also collected from the 9ml tubes of lysis buffer, and the silica supernates in the 2.0 ml screw-capped Eppendorf tubes. After chilling at 5 ^{o}C, supernates were mixed vigorously for 20 s with 200µl of Protein Precipitation Solution (SLS Ltd., UK) and centrifuged for 3min at 10,000g. Any pellet was discarded and 600µl isopropanol (−20 ^{o}C) added to 550µl of each supernate. Tubes were mixed by inversion 50 times and spun 3min. Supernates were discarded and tubes washed once with 500µl 70% ethanol (−20 ^{o}C). After draining, tubes were dried in a heating block. Any precipitated DNA was re-hydrated with 60µl elution buffer (NucliSens®), aliquoted and used immediately or stored at −20 ^{o}C. Negative extraction controls were processed in parallel with the test samples, with at least one negative control per seven samples.

DNA amplification and detection

Two specific regions of the *M. tuberculosis* complex were targeted – the repetitive elements IS*6110* (1–25 copies/cell) and IS*1081* (6 copies/cell). Initially DNA extracts were screened for the presence of *M. tuberculosis* DNA by conventional PCR (Table 1). Two specific regions found in all members of the *M. tuberculosis* complex, based on the insertion elements IS*6110* (Eisenach et al., 1990; Taylor et al., 1996) and IS*1081* (Taylor et al., 2003), which are normally multi-copy, were used to increase the likelihood of detection. In addition, nested or hemi-nested PCR, respectively, was used. PCR was carried out in 25 µl volumes using 5 µl of DNA extract, according to the parameters in Table1. Negative controls containing water were always run in parallel. Qiagen Hotstar® *Taq* polymerase and reagents (Qiagen, West

Sussex, UK) were used for first stage PCR reactions. Bovine serum albumin (BSA) at a final concentration of 10 mM was included in one set of reactions, as this has been shown to improve PCR yield (Forbes & Hicks, 1996; Abu Al-Soud & Rådström, 2000). Nested or hemi-nested PCR was performed using pre-aliquoted double-strength PCR mix (ABGene®, Surrey, UK). The final composition of the PCR mixture (50 µl) was 75 mM Tris/HCl (pH 8.8); 20 mM $(NH_4)_2SO_4$; 1.5 mM $MgCl_2$; 0.01% (v/v) Tween 20; 200 µM (each) dATP, dCTP, dGTP and dTTP; and 1.25 units *Taq* DNA polymerase. The primer pair and DNA preparation (1.0 µl) were added to each pre-aliquoted tube plus sufficient water to bring the volume to 50µl. Primer concentrations were 200nM.

Table 1: Primer sequences and conventional PCR details[1]

Locus	Primers (5' - 3')	$MgCl_2$ (mM)	Annealing temp. (^oC)	Product (bp)
IS*6110* Outer	P1: CTCGTCCAGCGCCGCTTCGG<br>P2: CCTGCGAGCGTAGGCGTCGG	1.5	68	123
IS*6110* Nested	IS3: TTCGGACCACCAGCACCTAA<br>IS4: TCGGTGACAAAGGCCACGTA	1.5	58	92
IS*1081* Outer	F2: CTGCTCTCGACGTTCATCGCCG<br>R2: GGCACGGGTGTCGAAATCACG	1.5	58	135
IS*1081* Hemi-nested	F2: CTGCTCTCGACGTTCATCGCCG<br>R3: TGGCGGTAGCCGTTGCGC	2.0	58	113

[1] An initial denaturation step (95°C for 15 min – hot start PCR); DNA amplification (initially 40 cycles, with 25 cycles in nested reactions) of strand separation at 94°C DNA for 40 sec, 1 min of primer annealing, followed by strand extension at72 ^{o}C for 20 sec plus 1 sec/cycle; and a final extension step of 1 min at 72 ^{o}C, were used for all PCR amplifications

In later experiments PCR was performed in a final volume of 25µl using a RotorGene© 3000 (Qiagen) real-time platform (Taylor et al., 2007). Qiagen Quantitect® SYBR Green PCR mix was used without BSA, but with additional Hotstar® *Taq* polymerase . Also, an annealing temperature of 60°C was used. The inclusion of the non-specific fluorescent reagent SYBR Green enables direct observation on screen of double-stranded amplified DNA. The determination of cycle threshold (Ct) indicates relative concentration of template. Amplicons are heat-degraded at the end of the run to determine the melting point (T_m).

References

Abu Al-Soud, W., Rådström, P., 2000. Effects of amplification facilitators on diagnostic PCR in the presence of blood, feces and meat. J. Clin. Microbiol. 38, 4463–4470.

Boom, R., Sol, C.J.A., Salimans, M.M.M., Jansen, C.L., Wertheim-van Dillen, P.M.E. et al. (1990) Rapid and simple method for purification of nucleic acids. J. Clin. Microbiol. 28: 495-503.

Eisenach, K.D., Cave, M.D., Bates, J.H., Crawford, J.T. (1990) Polymerase chain reaction amplification of a repetitive DNA sequence specific for *Mycobacterium tuberculosis*. J. Infect. Dis. 161: 977-981.

Forbes, B.A., Hicks, K.E., 1996. Substances interfering with direct detection of *Mycobacterium tuberculosis* in clinical specimen by PCR: effects of bovine serum albumin. J. Clin. Microbiol. 34, 2125–2128.

Höss, M., Pääbo, S. (1993) DNA extraction from Pleistocene bones by a silica-based purification method. Nucleic Acids Res. 21: 3913-3914.

Spigelman, M. Matheson, C., Lev, M. Greenblatt, C., Donoghue, H.D. (2002) Confirmation of the presence of *Mycobacterium tuberculosis* complex-specific DNA in three archaeological specimens. Int. J. Osteoarchaeol. 12: 393-401.

Taylor GM, Crossey M, Saldanha J, Waldron T (1996) *Mycobacterium tuberculosis* identified in mediaeval human skeletal remains using polymerase chain reaction. J. Archaeol. Sci. 23: 789-798.

Taylor GM, Stewart GR, Cooke M, Chaplin S, Ladva S et al. (2003) Koch's Bacillus – a look at the first isolate of *Mycobacterium tuberculosis* from a modern perspective. Microbiology 149: 3213-3220.

Taylor, G.M., Worth, D.R., Palmer, S., Jahans, K., Hewinson, R.G., 2007. Rapid detection of *Mycobacterium bovis* DNA in cattle lymph nodes with visible lesions using PCR. BMC Vet. Res. 3, 12–22.

Taylor, G.M., Mays, S.A., Huggett, J.F., 2010. Ancient DNA (aDNA) studies of man and microbes: general similarities, specific differences. Int. J. Osteoarchaeol. 20, 747–751.
