# Supplementary material for: Osteological and Biomolecular Evidence of a 7000-Year-Old Case of Hypertrophic Pulmonary Osteopathy Secondary to Tuberculosis from Neolithic Hungary
Source: PLoS One. 2013 Oct 30;8(10):e78252. doi: 10.1371/journal.pone.0078252 (PMC3813517; doi:10.1371/journal.pone.0078252)
Supplement: Document S3 — Detailed information on the lipid biomarker analysis. (PDF) [file pone.0078252.s003.pdf]

S3: Detailed information on the lipid biomarker analysis

The rib sample from HGO-53 (556 mg) was hydrolysed by heating with 30% potassium hydroxide in methanol (2 ml) and toluene (1 ml) at 100^0C overnight [1,2,3]. In a separate parallel experiment, standard biomass from *M. tuberculosis* was processed. Long-chain compounds were extracted as described previously [1,3] and the extract was treated with pentafluorobenzyl bromide, under phase-transfer conditions [1,2,3], to convert acidic components into pentafluorobenzyl (PFB) esters. Subsequent separation on an Alltech 209250 (500 mg) normal phase silica gel cartridge gave fractions containing non-hydroxylated fatty acid PFB esters, mycolic acid (MA) PFB esters and free phthiocerols [1,3].

The MA PFB esters were reacted with pyrenebutyric acid (PBA) to produce PBA-PFB MA derivatives, which were purified on an Alltech 205250 (500 mg) C_{18} reverse phase cartridge [1,3]. The PBA-PFB mycolates were analysed by sequential reverse and normal phase HPLC, as described previously [1,3]. The non-hydroxylated PFB esters were fractionated on an Alltech 205250 (500mg) reverse phase silica gel cartridge, using a water-methanol/methanol/methanol-toluene elution sequence [3]. A fraction enriched in mycocerosic acid and other longer chain (> C_{20}) PFB esters was eluted by 100% methanol with the more usual C_{12} to C_{20} esters eluting in the earlier water/methanol fractions. The fractions containing possible mycocerosates were analysed by negative ion chemical ionization gas chromatography mass spectrometry (NICI-GCMS), as previously described [2,3]. PFB esters, on NICI-GCMS, fragment to produce negative carboxylate $[M – H]^-$ ions, which can be detected at high sensitivity. Selected ion monitoring (SIM) was used to search for mycocerosate carboxylate ions at *m/z* 367.6311 (C_{24}), 395.6844 (C_{26}), 409.7111 (C_{27}), 437.7645 (C_{29}), 451.7911 (C_{30}), 479.8445 (C_{32}), 493.8712 (C_{33}) and 507.8978 (C_{34}). Additionally, *m/z* 407.6952 was monitored for the presence of the C_{27} mycolipenate carboxylate ion [2,3]. Partial racemisation of mycocerosates during the alkaline hydrolysis leads to the formation of diasteroisomers, which resolve on gas chromatography to give characteristic doublets; in contrast, mycolipenates are singlets as they cannot racemise [2,3].

References

1. Hershkovitz I, Donoghue HD, Minnikin DE, Besra GS, Lee OY-C, et al. (2008) Detection and Molecular Characterization of 9000-Year-Old *Mycobacterium tuberculosis* from a Neolithic Settlement in the Eastern Mediterranean. PLoS One 3: e3426.
2. Redman JE, Shaw MJ, Mallet AI, Santos AL, Roberts C, et al. (2009) Mycocerosic acid biomarkers for the diagnosis of tuberculosis in the Coimbra Skeletal Collection. Tuberculosis 89: 267-277.
3. Lee OY-C, Wu HHT, Donoghue HD, Spigelman M, Greenblatt CL, et al. (2012) *Mycobacterium tuberculosis* Complex Lipid Virulence Factors Preserved in the 17,000-Year-Old Skeleton of an Extinct Bison, *Bison antiquus*. PLoS One 7: e41923.
