# Supplementary material for: Osteological and Biomolecular Evidence of a 7000-Year-Old Case of Hypertrophic Pulmonary Osteopathy Secondary to Tuberculosis from Neolithic Hungary
Source: PLoS One. 2013 Oct 30;8(10):e78252. doi: 10.1371/journal.pone.0078252 (PMC3813517; doi:10.1371/journal.pone.0078252)
Supplement: Document S4 — Results of aDNA analysis - gels and melt. (PDF) [file pone.0078252.s004.pdf]

S4: Results of aDNA analysis

1. Gel electrophoresis of IS*1081* amplicons following conventional PCR

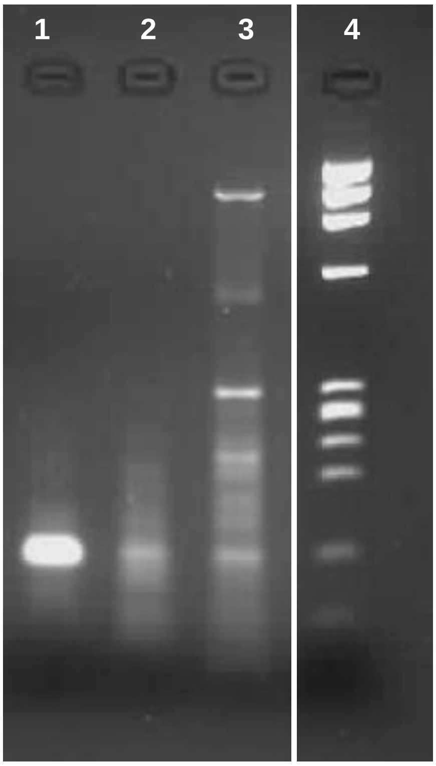

PCR amplicons using IS*1081* primers (113 bp): Lane 1 sample 225 (18th century Vác, Hungary); Lane 2: HGO53 vertebra; Lane 3: HGO53 vertebra extracted with PTB; Lane 4: molecular size markers (ΦX174 *Hae*III digest, band 2 is 118bp)

2. Gel electrophoresis of IS *1081* amplicons following real time PCR with SYBR Green

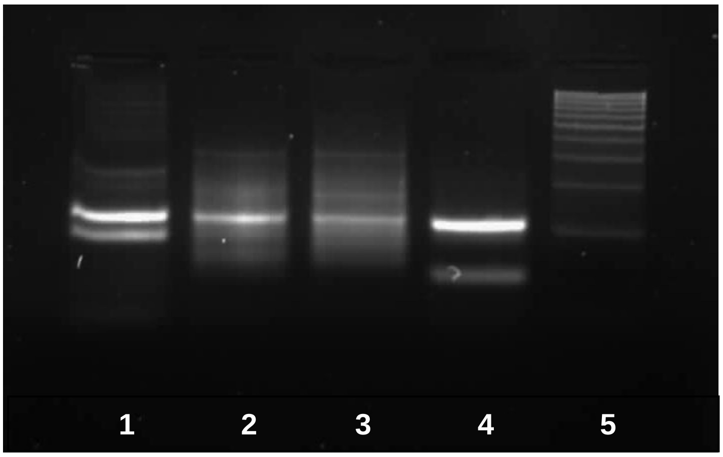

PCR amplicons using IS*1081* primers (113 bp): Lane 1 sample 225 (18th century Vác, Hungary); Lane 2: HGO53 vertebra; Lane 3: HGO53 vertebra extracted with PTB; Lane 4: sample 50 (Zalavár-Vársziget, Hungary); Lane 5: molecular size markers (20bp and 100bp ladders)

3. Melt analysis following IS*1081* PCR to determine T_m of amplicon

Profile

Cycle	Cycle Point
Hold @ 95°c, 15 min 0 secs	
Cycling (45 repeats)	Step 1 @ 95°c, hold 10 secs
	Step 2 @ 60°c, hold 30 secs
	Step 3 @ 72°c, hold 16 secs
	Step 4 @ 85°c, hold 15 secs, acquiring to Cycling A(FAM)
Melt (76-98°c) , hold 30 secs on the 1st step, hold 5 secs on next steps, Melt A(FAM)	

Melt data for Melt A.FAM

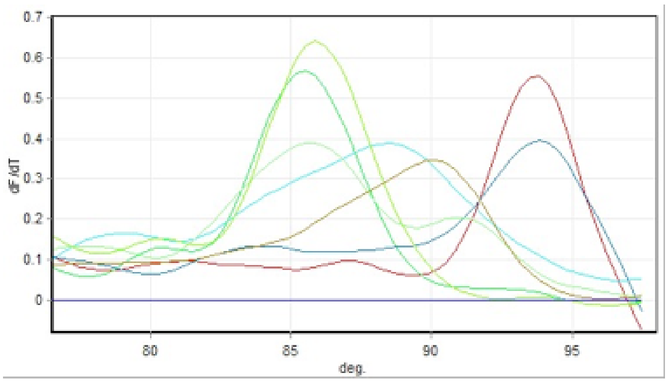

No.	Colour	Name	Genotype	Peak 1	Peak 2	Peak 3
1		225R+		81.3	87	93.7
2		V		79	88.5	
3		oEC		80.5	85.5	
4		V+		84	93.8	
5		oEC+		77.7	85.7	91
13		EC		80.5	85.8	93.8
21		PCRC		90		

No. 1 – sample 225 (18th century Vác, Hungary)
No. 2 – sample HGO-53 (vertebra) extracted without PTB
No. 3 – original negative extraction control extracted without PTB
No. 4 – sample HGO-53 (vertebra) extracted with PTB
No. 5 – original extraction control extracted with PTB
No.13– extraction control for later batch without PTB
No.21– water blank reaction negative control
[Other channels used for later batch of different samples so are not shown]

The T_m of the positive control (No. 1) is 93.7 ^{o}C and for the HGO-53 vertebra (No. 4) is 93.8 ^{o}C. This is an acceptable match but requires DNA sequencing for confirmation.

The sequencing did not give acceptable data so lipid molecular markers were sought in order to confirm the provisional identification of *Mycobacterium tuberculosis* complex DNA.
